# Development of a nursing-specific Mini-CEX and evaluation of the core competencies of new nurses in postgraduate year training programs in Taiwan

**DOI:** 10.1186/s12909-019-1705-9

**Published:** 2019-07-18

**Authors:** Yueh-Ping Liu, Dana Jensen, Cho-yu Chan, Chung-jen Wei, Yuanmay Chang, Chih-Hsiung Wu, Chiung-hsuan Chiu

**Affiliations:** 1 Department of Emergency Medicine, National Taiwan University Hospital, Taipei, Taiwan; 2 School of Health Care Administration, Taipei Medical University, 250 Wu-hsing St., Taipei, Taiwan; 3 Center for Teaching Excellence, Changhua Christian Hospital, Changhua City, Taiwan; 4 Department of Public Health, Fu Jen Catholic University, New Taipei City, Taiwan; 5 Institute of Long Term Care, MacKay Medical College, New Taipei City, Taiwan; 6 College of Medicine, Taipei Medical University, Taipei, Taiwan

**Keywords:** Nursing-specific mini-CEX, New nurses, Post-graduate year program

## Abstract

**Background:**

Modern nursing requires a broad set of academic and practical skills, and an effective nurse must integrate these skills in a wide range of healthcare contexts. Cultivation of core competencies has recently become a key issue globally in the development of nursing education. To assess the performance of new nurses, this study developed a nursing-specific Mini-Clinical Evaluation Exercise (Mini-CEX) to evaluate the effect of postgraduate year (PGY) nurse training programs in Taiwan.

**Methods:**

A nursing-specific Mini-CEX was developed based on the required core competencies of nurses. Reliability and validity were confirmed in evaluator workshops carried out prior to the administration of the pilot test and final test. Thirty-two PYG trainees were recruited with a supervisor-to-trainee ratio of 1:1.94. Data were collected from February to June 2012 and analyzed using the Kruskal-Wallis test.

**Results:**

The 32 PGY trainees scored highest in the “nursing professionalism” dimension and the lowest in the “physical examination” dimension. The overall competency score was satisfactory. The trainee nurses with 19–24 months of experience scored higher than the other two groups in overall performance.

**Conclusion:**

The results of this research indicate the feasibility of using our Mini-CEX tool to evaluate the competencies of PGY trainees.

**Electronic supplementary material:**

The online version of this article (10.1186/s12909-019-1705-9) contains supplementary material, which is available to authorized users.

## Background

Modern nursing requires a broad set of academic and practical skills, and an effective nurse must integrate these skills in a wide range of healthcare contexts [1]. New nursing school graduates often find the transition into independent clinical practice challenging, especially in the first year. Studies show that they often struggle to prioritize patient care, recognize and manage patient problems, understand the rationale for approaches to these problems, and communicate constructively with both the care team and patients [1, 2]. Many studies have also documented the efficacy of well-designed nurse transition programs provided in some European and North American countries in improving new graduates’ clinical competence [3]. Cultivation of core competencies has recently become a key issue in the development of nursing education globally.

To help equip postgraduate year (PGY) trainees with the necessary competencies, three issues must be addressed. Firstly, the core competencies must be defined based on established definitions. Secondly, effective methods to bridge the gap between academic knowledge and clinical practice must be developed. Thirdly, effective performance evaluations must be established.

Nurses learn their core competencies from their role relationships with other medical practitioners, socialization, acculturation to nursing practice, and acquisition of the knowledge embedded in practice [4]. There is no consensus on the specific definitions of core competencies necessary to perform clinical tasks, and the required competencies vary according to the work site, unit, and setting, resulting in very different experiences [5]. Therefore, instead of using a single source (i.e. a single theorist) to define core competencies, it is more appropriate to synthesize definitions from a range of sources. These should include the knowledge, skills, and practices needed to perform as a professional nurse; definitions from extant literature; general standards from nursing associations; and the social contract with the general public. Therefore, core competencies should cover the integration of knowledge, attitudes, and behaviors necessary to practice safely and with high standards of patient care in a clinical setting [6]. The “ladder system” of skill acquisition can help define the progress of nurses. According to the Dreyfus Model, modified from Benner, the ladder system establishes five stages of nursing proficiency (novice, advanced beginner, competent, proficient, and expert) [4]. Experiences are incorporated into the definitions of clinical competencies through advancing stages of practice. These stages also provide guidelines for expected progress [5].

Before taking the nursing certification exam and beginning PGY training, trainees must have either completed a four-year undergraduate degree after senior high school, a five-year associate degree after junior high school (which is often followed by a two-year undergraduate program), or a four-year undergraduate degree after vocational senior high school [7]. In addition to the academic curriculum, students must perform 1,120 h of clinical training: 60 h of nursing fundamentals; 240 h of medical-surgical nursing; 120 h each of maternal-newborn, pediatric, community health, and psychiatric nursing; and 340 additional hours in unspecified settings such as administration and senior practicums [8, 9]. The licensing examination is written, although objective structured clinical examinations (OSCEs) are to be added in the near future [10]. Nurses who are hired at teaching hospitals within 4 years of obtaining their license are required to enter PGY training programs.

To ensure the quality of healthcare in Taiwan, the Ministry of Health and Welfare (MOHW) has adopted the World Federation for Medical Education’s guidelines and the World Health Organization’s Framework for Action on Interprofessional Education and Collaborative Practice, which recommend using institutional medical education as a starting point for integrating education and training. The MOHW has also enacted the Teaching Quality Improvement Program for Teaching Hospitals, which has been helping medical and paramedical professionals establish postgraduate clinical training systems since 2007 [11]. The MOHW also supports and reimburses teaching hospitals for postgraduate healthcare staff training through the “Instruction Fee Reimbursement Programs for Teaching Hospitals.” The postgraduate nurse training program supported by the MOHW runs for 2 years. The first 3 months are comprised of location-based curriculum training followed by 9 months of core curriculum training and a year of professional training [11, 12]. The program aims to equip PGY trainees with (1) Professional nursing knowledge, the ability to provide quality care, evidence-based nursing skills, and resource management ability; (2) Patient-centered and total care attitudes and skills; (3) Professional ethical reasoning and communication skills; and (4) The ability to work in a team [13]. Other major professional, regulatory, and statutory organizations have introduced similar programs to help new nurses bridge the gap between academic knowledge and clinical performance, such as the Scottish Flying Start program [14] and the Transition to Practice program (launched by the US National Council of State Boards of Nursing, or NCSBN) [15]. Recognizing that it is important to demonstrate the effectiveness of the two-year postgraduate training program to justify its substantial budget, the MOHW has been developing a comprehensive policy evaluation plan.

Competency assessment tools require rigorous confirmation of validity and reliability [5]. In addition to the self-reported assessment tool, other popular work-based assessment approaches include portfolios and OSCEs [16]. A few modifications to these two formats have been attempted, such as the transition from traditional assessments to workplace-based assessments; two examples are the Mini-Clinical Evaluation Exercise (Mini-CEX) and Direct Observation of Procedural Skills [17].

The Mini-CEX was proposed by the American Board of Internal Medicine in 1972 to address deficits in traditional clinical evaluations for residents. This new shortened evaluation format assesses a resident’s clinical judgement and patient counseling skills based on their ability to take a patient history and perform a physical exam. This Mini-CEX for medical students and residents reflects attending physicians’ expectations for teaching rounds, and purposefully focuses on the skills needed in an actual patient encounter. The patient encounters are approximately 15 to 20 min long, during which time the students or residents are rated from 1 to 9 in seven areas based on the skills demonstrated in the encounter [18]. The Mini-CEX scores are based on real and varied patient encounters as observed by experienced educator-clinicians, which provides validity and reliability to the assessment [19].

As defined by Virginia Henderson [20], “The unique function of the nurse is to assist the individual, sick or well, in the performance of those activities contributing to health or its recovery (or to a peaceful death) that he would perform unaided if he had the necessary strength, will or knowledge. And to do this in such a way as to help him gain independence as rapidly as possible” [21]. Although physicians and nurses share common ground on patient care, many of the required competencies, attitudes, and practices are different, especially in relation to the caring role of nurses [5, 21, 22]. Therefore, a customized measurement tool for nurses is required.

Due to growing awareness surrounding issues of nurse competence, developing a new nurse assessment has become increasingly important to educators and administrators. It is necessary for the maintenance of public safety and high standards for the profession [3, 23]. The utilization of a nursing-specific Mini-CEX is recommended in Taiwan because of its efficiency, reliability, validity, and practicality [24]. The purpose of this study was to develop a specialized Mini-CEX for nurses in order to evaluate the effectiveness of PGY training programs as a component of required evaluation in Taiwan.

## Methods

### Study design

**Fig. 1:**
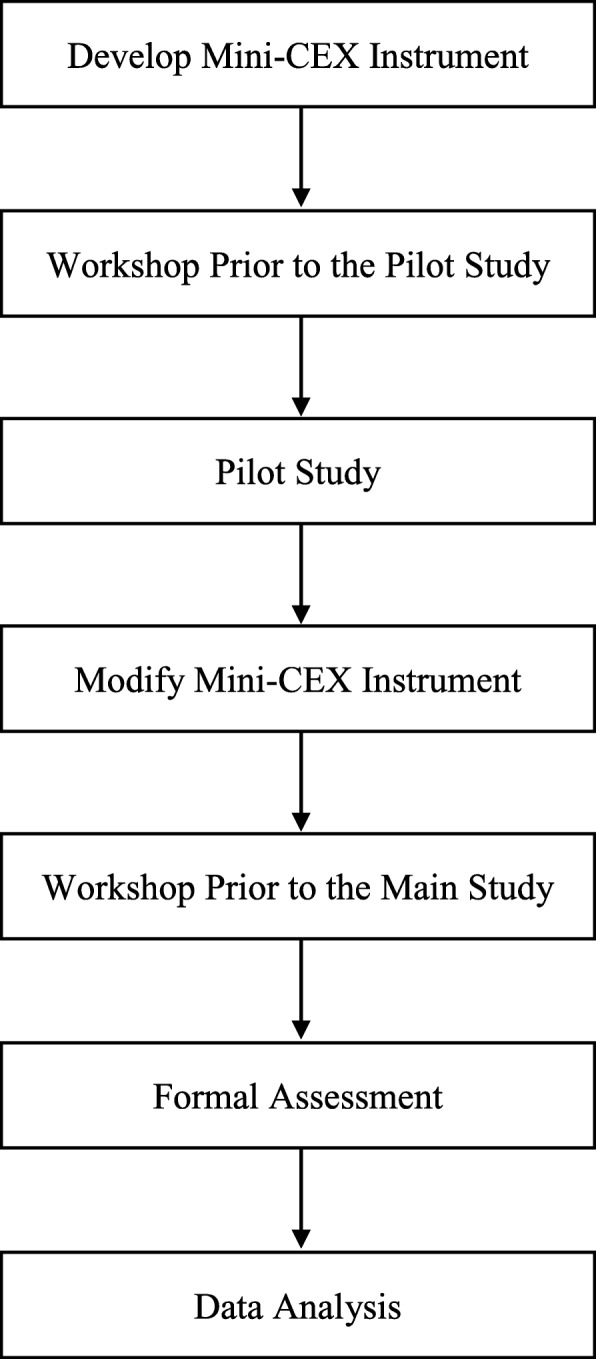
Study flowchart

The instrument was devised and tested in six phases: (1) The nursing-specific Mini-CEX instrument was developed according to nurses’ core competencies as defined by expert interviews and extant literature; (2) An initial workshop for evaluators was held prior to the pilot test to insure inter-rater reliability; (3) A pilot test on a group of new nurses was held to provide feedback for reevaluation; (4) Workshops were held for evaluators before the main study; (5) Cross-sectional observations were launched to measure the competencies of new nurses from the perspective of instructors; and (6) Descriptive statistics and the Kruskal-Wallis test were used to examine the PGY trainees’ Mini-CEX scores based on the duration of their training. The study flowchart is listed in Fig. 1.

### Instrument development

This study defined nurses’ core competencies as the skills, abilities, and knowledge needed to practice independently in a clinical setting [6, 25–27]. Since the depth and breadth of knowledge is very different for new and experienced nurses, this study focused on the professional development path of new nurses and included the criterion-related standards with which a “competent” nurse (level 2) is expected to be equipped [4, 21, 28].

In addition to the definition of core competencies provided by the Taiwan Nursing Accreditation Council (TNAC) [6, 25], this study also considered the core competencies of new nurses as defined by other entities, such as the Flying Start program [14], the Transition to Practice program [15], and the Canadian Nursing Association (CNA) [29]. Six core competency dimensions were selected for this study: medical knowledge and clinical skills, communication, teamwork, ethical consideration, policy and management, and public health duties (Table 1). Since the study targeted new nurses who had just started practicing in a clinical setting after passing their board examinations, we exclusively focused on “observable” competencies, such as behavior, skills, and attitudes.

**Table 1:** Core competencies of new nurses

Major Dimensions	TNAC	Transition to Practice Program (NCSBN)	Flying Start program (NHS)	Entry level nurse core competencies (CNA)
Medical Knowledge and Clinical skills	Basic biomedical knowledge			
	General clinical skills	Patient- and family-centered care	Clinical skills	
		Quality improvement	Safe practice	
		Evidence-based practice	Research for practice	Knowledge-based practice
		Informatics		
			Reflective practice	
	Critical thinking and reasoning			
	Caring			
	Lifelong learning		Professional development	
Communication	Communication capability	Communication	Communication	
Teamwork	Teamwork capability	Teamwork	Teamwork	
Ethical consideration	Ethics		Equality & diversity/ patient autonomy	Ethical practice
	Accountability			Professional responsibility and accountability
				Self-regulation
Policy and Management			Policy	
Public Health Duties				Service to the public

Note:

https://cna-aiic.ca/~/media/cna/files/en/clinical_nurse_specialists_convention_handout_e.pdf

https://www.nurses.ab.ca/docs/default-source/document-library/standards/entry-to-practice-competencies-for-the-registered-nurses-profession.pdf?sfvrsn=15c1005a_12

Based on the core competencies defined in this study, a Mini-CEX for nurses was developed to include observable skills, such as taking a patient’s history, giving a physical examination, managing intervention/therapy, exercising clinical judgment, counseling, professionalism, organization/efficiency, and overall competency (Table 2). The evaluation form also required detailed descriptions of each dimension and sample behavior items based on expert interviews, various nursing associations [4, 30, 31], and literature [32, 33].

**Table 2:** Cross-comparison of Mini-CEX items and core competencies

Major Dimensions	Medical Knowledge and Clinical Skills	Communication	Teamwork	Ethical Consideration	Policy and Management	Public Health Duties
History Taking	✓	✓		✓		
Physical Examination	✓			✓		
Intervention/ Therapeutic Skills	✓					
Counseling Skills	✓	✓				✓
Nursing Professionalism			✓	✓	✓	✓
Organization / Efficiency					✓	
Overall	✓	✓	✓	✓	✓	✓

### Validity and reliability check of the nurse-specific mini-CEX

The instrument was validated by eight experts: two MD/PhDs who specialize in developing evaluations, three RN/PhDs familiar with the methodology, and three directors of nursing. They assessed the feasibility and wording of the items and detailed descriptions. Two rounds of the Delphi method were applied to check the face validity and the allocation of sample behavior items to the appropriate dimensions [34]. In the first round, experts modified the wording of items and descriptions according to the definitions utilized in the study. The authors referenced the first-round comments from experts and their own observations to validate the items and descriptions in the second-round Delphi.

We utilized three ratings to decide whether or not to include dimensions: dimension is important and should be retained (3 points), dimension is important but needs revision (2 points), and dimension is not important and must be removed (1 point). We retained dimensions that scored an average of at least 2 points. Wording of items and descriptions was modified and rechecked if experts awarded 2 points.

The final version of the Mini-CEX tool included seven dimensions: history taking, physical examination, intervention/therapeutic management, counseling skills, nursing professionalism, organization/efficiency, and overall competency (see Additional file 1). All descriptive items for each dimension were modified based on guidelines provided by the CNA [30], which were translated into Chinese and adjusted to depict the standards for a “competent” nurse. Descriptions of each dimension are provided in Appendix.

Evaluators rated trainees based on the performance expected of a “competent” nurse. Each core competency was rated on a nine-point scale as unsatisfactory (1–3), satisfactory (4–6), or excellent (7–9), a system that has been proven to be more effective for trainee assessments than five-point scales [13]. Unsatisfactory ratings are defined as “extremely poor” (1 point), “poor” (2 points), and “nearly passing” (3 points). Satisfactory ratings are defined as “meets minimum expectations” (4 points), “average” (5 points), and “slightly above average” (6 points). Excellent ratings are defined as “meets most expectations and exceeds all others” (7 points), “exceeds most expectations and meets all others” (8 points), and “exceeds all expectations” (9 points). All behaviors are rated based on observed bedside practices for the selected case.

### Pilot study

To confirm the fitness of the instrument, a convenience sample was selected and a pilot test was conducted at two hospitals in Taipei in December 2011. Six PGY trainees and six evaluators were recruited. The inclusion criteria for trainees and evaluators are listed below, and were applied to both the pilot study and the main study. Trainee nurses were required to have a nursing license, be participating in a PGY program (for fewer than 2 years at the time of the study), and be employed in a general internal medicine ward of one of the three teaching hospitals included in the study. Evaluators were all qualified nurses in the hospital staff with instructor certification. To receive instructor certification, nurses are required to attend at least 10 h of faculty development programs, fulfill the teaching tasks assigned by their hospitals, and pass the accreditation examination held by the Joint Commission of Taiwan. All six trainees were women with a mean age of 21.67 (SD 5.69) and mean tenure of 2.07 months (SD 2.06).

A workshop was held for the evaluators prior to the pilot test to reach a consensus on the standards of scoring and to maintain inter-rater reliability. The workshop was divided into two sessions. In the first session, the background, concept, purpose, and procedure of the nursing-specific Mini-CEX were presented. In the second session, a video of a nursing-specific Mini-CEX encounter was shown to the evaluators, who were then asked to score the encounter and briefly explain their reasoning to the other evaluators. They then discussed their definitions of “excellent,” “satisfactory,” and “unsatisfactory” before the moderator led a group discussion to reach a consensus on evaluation definitions, observed behaviors, and the content of the observed scenario. Once the evaluators reached a consensus on the scoring standards, the video was shown again to the evaluators for scoring. Evaluators were advised to complete the observation with “sandwich” feedback followed by direct instructions for improvement [35].

To check the consistency of the instrument, we collected scores from both rounds of the evaluator workshop. A first-round evaluation was collected after the clip played. We then obtained a second evaluation after an instructor-hosted discussion in which a consensus was reached among the evaluators on the standard of performance shown in the video. We obtained the inter-rater reliability of this instrument on the basis of the second evaluation. The inter-rater reliability was 0.7.

### Main study

Since this study focused on the assessment of trainees based on the judgements of evaluators, we recruited two independent groups of evaluators and trainees. For the main study, PGY trainees and senior nurse instructors were recruited from the general wards of three teaching hospitals in Taipei between February and June 2012. Seventeen evaluators and 32 trainees participated, giving an evaluator to trainee ratio of 1:1.94.

A workshop to enhance inter-rater reliability was attended by all selected instructors before the main study began. The protocol was identical to that of the pilot study workshop.

### Ethical consideration

The Joint Institutional Review Board of Taipei Medical University approved the study. The approval number is TMU 201012008. Written informed consent was obtained from the faculty members and PGY trainees after they received an explanation of the goals and purposes of the study and were assured that their assessments would be confidential.

### Statistical analysis

The results were analyzed using SPSS for Windows 22.0 in three parts. Firstly, the inter-rater reliability was calculated based on the scores given by the evaluators before and after the discussion in the workshop session. Secondly, descriptive statistics and the Kruskal-Wallis test were used to examine the PGY trainees’ Mini-CEX scores based on the duration of their training.

## Results

### Descriptive statistics

A total of 32 PGY trainees and 17 evaluators participated. The study reached 40 PGY trainees and collected 32 questionnaires. Trainees were recruited from three hospitals (10, 13, and 9 respectively) and were divided into three groups according to duration of training: 18 trainees (56.5%) in the 4–12 month group, 6 trainees (18.8%) in the 13–18 month group, and 8 trainees in the 19–24 month group. The average duration of training for the entire sample group was 12.69 months. They were all women (100%) and their mean age was 23.3 (SD 1.75), ranging from 20 to 24 years (75%) and 25 to 29 years (25%).

The trainees scored highest in the “nursing professionalism” domain with a mean score of 6.56 (SD 1.19), and the lowest in the “physical examination” domain with a mean score of 6.17 (SD 1.34). The mean overall competency score was 6.53 (SD 1.14), meaning that the competence of trainees enrolled in the study met our expectations (Table 3). In the subgroup analysis, trainees with 4–12 months of PGY training obtained their highest scores in “nursing professionalism” (mea*n* = 6.39, SD = 1.09), while trainees with 19–24 months of PGY training scored highest in “intervention/therapeutic management” with a mean score of 7.38 (SD = 1.06). On average, trainees with 19–24 months of PGY training obtained higher scores than less-experienced trainees in all domains except for “counseling skills.” However, no statistically significant difference was indicated between the performances of the four groups (Table 3).

**Table 3:** Cross-comparison of evaluation scores by trainee group (Kruskal-Wallis test)

Domains	Participants (duration of training)				*p* value
	Total (*N* = 32)	4–12 months (*n* = 18)	13–18 months (*n* = 6)	19–24 months (*n* = 8)	
History Taking	6.30 (1.15)	6.12 (1.05)	6.00 (0.89)	7.00 (1.41)	0.29
Physical Examination	6.17 (1.34)	5.81 (1.33)	5.83 (1.17)	7.13 (1.13)	0.08
Intervention/Therapeutic Management	6.47 (1.32)	6.17 (1.30)	6.17 (1.33)	7.38 (1.06)	0.09
Counseling Skills	6.25 (1.24)	6.28 (1.18)	6.00 (0.89)	6.38 (1.69)	0.71
Nursing Professionalism	6.56 (1.19)	6.39 (1.09)	6.33 (1.21)	7.13 (1.36)	0.35
Organization/Efficiency	6.42 (1.21)	6.18 (1.24)	6.17 (0.75)	7.13 (1.25)	0.15
Overall	6.53 (1.14)	6.28 (1.80)	6.33 (0.82)	7.25 (1.04)	0.16

## Discussion

Considering the need for healthcare professionals to have both academic knowledge and practical skills, it is especially important for their training to integrate both of these elements. This need for effective training applies to both pre-employment training as well as continuing education [36, 37]. As the attention being paid to quality of care and practitioner competency increases, the need for tools to evaluate these competencies is growing. The literature indicates that work-based formative assessments can have a substantial impact on learners’ behaviors [38–40]. To these aims, this study customized the Mini-CEX assessment tool for nurses by integrating expert opinions, clinical guidelines, and published literature [32]. This process produced seven main domains for PGY trainee assessment: history taking, physical examination, intervention/therapeutic skills, counseling skills, nursing professionalism, organization/efficiency, and overall competency. The seven items address the expectations of internal forces, such as nursing associations, and external forces, such as hospitals, governments, patients, and the general public [4]. To assist new nurses in developing their clinical competencies, clear guidelines and a systematic curriculum are crucial. This Mini-CEX provides a tool to evaluate trainees’ abilities to meet the requirements set by internal and external forces, as well as a simple method for mentors to observe and reinforce desired behaviors.

### Instrument validity and reliability

The instrument was checked for inter-rater reliability to confirm the consistency of evaluation scores, and the two-round Delphi method was used to confirm the face validity [40]. Evaluation scores primarily ranged from 4 (satisfactory) to 9 (excellent), and very few trainees received evaluations under 3 (unsatisfactory). The authors cross-checked the scores with the comments given by evaluators, and the scores awarded were found to be consistent with the comments. For example, one trainee with 22 months of training was given a 7 in history taking, 8 in physical examination, 7 in intervention/therapeutic skills, 7 in counseling skill, 8 in professionalism, 8 in organization/efficiency, and 8 in overall performance. She earned comments such as “showed concern for patient’s feedback, very good performance of sterilization procedures. Overall performance is good.” Another trainee with 8 months of training was given a 5 in history taking, 5 in physical examination, 4 in intervention/therapeutic skills, 5 in counseling skill, 5 in professionalism, 5 in organization/efficiency, and 5 in overall performance, with comments such as “trainee is able to perform history taking and provide counseling to the patient.” This narrative feedback echoed the scores given by evaluators.

### Sample demographic

In this study, all trainee participants were women. Approximately 2% of the professionally active nursing workforce in Taiwan is male, according to December 2015 data from the Taiwan Union of Nurses Association [41]. The imbalance might be extreme, but it reflects the global reality.

### Major findings

We found that 4–12 month trainees obtained lower scores in most domains than 19–24 month trainees, especially “physical examination,” although no statistically significant difference between the two groups was noted. The 19–24 month trainees may have had more opportunities to practice clinical assessments (including physical examinations) on patients, possibly explaining why the 19–24 trainees performed better. This suggests that while PGY trainees are expected to be appropriately skilled in giving physical examinations, they may not have been sufficiently educated in these skills at school. Nurses and other healthcare professionals utilize inspection, palpation, percussion, and auscultation to assess their patients’ health status and contextualize subjective data to guide their clinical decisions. These techniques are a necessary source of clinical data and their application is often guided by patient or provider concerns in the form of a problem-focused physical examination [42]. All nursing students in Taiwan study physical assessment as a major component of their curriculum, but this may not translate optimally into practice.

A Mini-CEX is expected to be a 20-min encounter during which a trainee takes a patient’s history and performs a physical examination while a faculty assessor observes. After the trainee discusses the diagnosis and management plan with the patient, the faculty member assesses the trainee using the Mini-CEX evaluation form and provides feedback [43]. In this study, we designed a nursing-specific version of the Mini-CEX that was well received by evaluators as an easy-to-use tool for assessing various nursing competencies. There were 17 evaluators in this study with acceptable reliability (inter-rater reliability = 0.7). Perhaps because other programs in Taiwan have trained evaluators to utilize the Mini-CEX as a formative assessment tool since 2009, all of our participating evaluators had already received similar training. This finding is consistent with previous studies that indicate the benefits of faculty development programs and rater training programs to the usefulness of the Mini-CEX [44].

As higher scores on the nursing-specific Mini-CEX were associated with more PGY training experience, this study has demonstrated that it is feasible to use the nursing-specific Mini-CEX to assess PGY trainees’ professional development. However, the results of a single Mini-CEX, especially if it is the only method of assessment, may not be able to fully demonstrate all of a trainee’s competencies. The core purpose of the Mini-CEX is to provide trainees with immediate and structured feedback based on observed performance. Several conditions are required for formative assessment strategies to create optimal positive change: Criteria for success must be clear, feedback must be immediately available following the assessment, the assessment must be a cohesive part of the learning process, and there must be multiple opportunities for assessment [38]. Performance is not solely motivated by internal factors. Organizational and social factors, such as resource availability, clear performance standards, and positive incentivization, are all tied to improved performance [45]. The hidden curriculum is intrinsically linked to these and other factors in the training environment [44]. Thus, it is important to establish organizational approaches to the cultivation of professionalism in PGY nurse training programs.

### Limitations

This study had five primary limitations. Firstly, it was a preliminary study with relatively few participants over a limited period of time. The results may be not generalizable to other settings, because the institutional environment and leadership are important determinants of a successful long-term formative assessment program. Secondly, the changes in clinical practice behaviors following evaluation and feedback were not measured. Changes in behavior can be influenced by many individual and external factors. It is necessary to derive strategies that involve the regulatory, educational, and practice components of nursing to ensure that PGY trainees use comprehensive methods to plan and monitor patients’ healthcare. This will in turn enhance quality of care. Thirdly, leniency error [46] possibly occurred, even though the research team enhanced the inter-rater reliability before launching the assessment. Fourthly, the nursing-specific Mini-CEX is a discrete, work-based assessment tool, while nursing practice requires ongoing and holistic patient care. Therefore, it may be better to use this instrument as one aspect of a global assessment instead of as the sole assessment tool. Finally, data were collected based on cross-sectional observations due to time limitations. Ideally it would be possible to analyze and track the educational impact of participating in this assessment. Understanding the effects of participation would enable the assessment to be integrated into the curriculum to influence positive change [47]. Further studies assessing changes in clinical competence over the course of the PGY programs and the effects of evaluations on nursing education are needed [23].

## Conclusion

For this study, the authors produced seven main dimensions for PGY trainees’ assessment: history taking, physical examination, intervention/therapeutic skills, counseling skills, nursing professionalism, organization/efficiency, and overall competency. We believe that these modifications have created an evaluation tool that is more compatible with the core values of nursing. Additionally, it is a practical tool for nurse-educators to use in workplace-based assessments. Supervisors can easily use this tool to evaluate a trainee’s strengths and weaknesses, and to give timely formative feedback. Nevertheless, further studies using this tool are recommended to address the effects of evaluation in changing practice behaviors.

### Additional file

Additional file 1: Mini-Clinical Evaluation Exercise (Mini-CEX) for Nurses. (DOCX 25 kb)

## Data Availability

No data have been submitted to any open-access databases. All data supporting the study are presented in the manuscript or are available upon request.

## Appendix

### Definitions of the seven dimensions

#### History Taking

*This* Table 3 of consideration for the needs of the patient and the patient’s current stage of life. It also addresses the nurse’s ability to perform a focused and comprehensive health assessment as appropriate for a given patient, and to generate an accurate record of the patient’s history.

#### Physical Examination

This dimension addresses a trainee’s ability to perform a complete physical examination and to explain and interpret the normal/abnormal results.

#### Intervention/Therapeutic Management

This dimension addresses whether a trainee considers the patient’s health condition when performing intervention/therapy, assesses the risk and cost of potential options, and ultimately chooses the appropriate intervention/therapy. When performing a nursing intervention/therapy, A trainee must obtain the patient’s informed consent. A nurse’s preparation, skills while performing an intervention/therapy, and procedures after the intervention/therapy must be appropriately skillful to ensure the best possible patient outcome.

#### Counseling Skills

This dimension assesses a trainee’s ability to discuss a drug therapy’s effects and side effects, potential drug interactions, the importance of medication compliance, and the recommended follow-up schedule with the patient during a drug consultation. A trainee can consult with the patient on ways to promote good health, provide group or individual health education, and provide support in planning and reviewing the patient’s care plan prior to discharge.

#### Nursing Professionalism

The nursing professionalism dimension assesses a nurse’s ability to perform according to the law, as well as the professional and ethical standards and policies of nursing professionals. The trainee must strictly protect the patient’s privacy and perform according to local laws, health policies, and standards.

#### Organization/Efficiency

This section tests a trainee’s ability to synthesize clinical decisions, resource allocation concerns, and cost-effectiveness principles. It also addresses whether the diagnosis and procedure process are logical and efficient.

#### Overall Competency

This section tests the overall competency of a trainee.

## Abbreviations

CNA: Canadian Nursing Association
Mini-CEX: Mini-Clinical Evaluation Exercise
MOHW: Ministry of Health and Welfare
NCSBN: National Council of State Boards of Nursing
NHS: National Health Service
OSCEs: Objective structured clinical examinations
TNAC: Taiwan Nursing Accreditation Council

## References

[1] Blanzola C, Lindeman R, King ML (2004). Nurse internship pathway to clinical comfort, confidence, and competency. J Nurses Staff Dev.

[2] del Bueno D (2005). A crisis in critical thinking. Nurs Educ Perspect.

[3] McMullan M, Endacott R, Gray MA, Jasper M, Miller CM, Scholes J (2003). Portfolios and assessment of competence: a review of the literature. J Adv Nurs.

[4] Benner P (1984). From novice to expert: excellence and power in clinical nursing practice.

[5] Watson R, Stimpson A, Topping A, Porock D (2002). Clinical competence assessment in nursing: a systematic review of the literature. J Adv Nurs.

[6] Hsu LL, Hsieh SI (2009). Testing of a measurement model for baccalaureate nursing students’ self-evaluation of core competencies. J Adv Nurs.

[7] Lu MS. Nursing education in Taiwan: the current situation and prospects for the future. J of Nurs. 2004;51(4):11-17. (in Chinese). 15290636

[8] Examination Yuan of R.O.C. Internship criteria for professional and technical examination of registered professional nurse by civil service senior examination and registered nurse by civil service junior examination. Taiwan: Examination Yuan of R.O.C. 2012. http://weblaw.exam.gov.tw/Attachment/Law/D03020005015/A070040120006100-1030304-6000-017-%E5%B0%88%E9%96%80%E8%81%B7%E6%A5%AD%E5%8F%8A%E6%8A%80%E8%A1%93%E4%BA%BA%E5%93%A1%E9%AB%98%E7%AD%89%E8%80%83%E8%A9%A6%E8%AD%B7%E7%90%86%E5%B8%AB%E8%80%83%E8%A9%A6%E3%80%81%E6%99%AE%E9%80%9A%E8%80%83%E8%A9%A6%E8%AD%B7%E5%A3%AB%E8%80%83%E8%A9%A6%E5%AF%A6%E7%BF%92%E8%AA%8D%E5%AE%9A%E5%9F%BA%E6%BA%96.pdf. Accessed 29 Oct 2012.

[9] Yang WP, Chao CSC, Lai WS, Chen CH, Shih YL, Chiu GL (2013). Building a bridge for nursing education and clinical care in Taiwan—using action research and Confucian tradition to close the gap. Nurse Educ Today.

[10] Ministry of Examination R.O.C. The feasibility seminar for the inclusion of the clinical skills test (OSCE) in the National Examination for nursing was completed successfully. 2011. https://wwwc.moex.gov.tw/pda/news/wfrmNews.aspx?kind=3&menu_id=1111&news_id=930. Accessed 4 Apr 2019.

[11] Yin YC (2013). The two-year post graduate training program for nurses: implementation status and personal perspectives. Hu Li Za Zhi..

[12] Joint Commission of Taiwan. Joint Commission of Taiwan. 2012. http://www.tjcha.org.tw/FrontStage/page.aspx?ID=5DDE57A5-EE32-47DC-B4EE-BD70E32522F7&PID=F119DC8B-752D-4971-A868-C109A44118C5. Accessed 29 Oct 2012.

[13] Cook DA, Beckman TJ (2009). Does scale length matter? A comparison of nine-versus five-point rating scales for the mini-CEX. Adv Health Sci Educ Theory Pract..

[14] Flying Start NHS. Flying Start: Developing confident and capable health practitioners. https://learn.nes.nhs.scot/735/flying-start-nhs. Accessed 16 July 2019.

[15] National Council of State Boards of Nursing (2018). Transition to Practice Program National Council of State Boards of Nursing.

[16] O'Connor SE, Pearce J, Smith RL, Voegeli D, Walton P (2001). An evaluation of the clinical performance of newly qualified nurses: a competency based assessment. Nurse Educ Today.

[17] Hassan S (2012). Task integrated objective structured clinical examination (TIOSCE): a modified version of OSCE. Educ Med J.

[18] Al Ansari A, Ali SK, Donnon T (2013). The construct and criterion validity of the mini-CEX: a meta-analysis of the published research. Acad Med.

[19] Norcini JJ (2005). The mini clinical evaluation exercise (mini-CEX). Clin Teach.

[20] Henderson V (1966). The nature of nursing a definition and its implications for practice, research, and education.

[21] Sweet LP, Glover P, McPhee T (2013). The midwifery miniCEX–A valuable clinical assessment tool for midwifery education. Nurse Educ Pract.

[22] Royal College of General Practitioners (2018). Clinical Evaluation Exercise (MiniCEX).

[23] Chen YH, Watson R (2011). A review of clinical competence assessment in nursing. Nurse Educ Today.

[24] Walter C, Lin CC, Ching HC, Tsai CH, Tsai CH (2006). Implementation of the mini-CEX (clinical evaluation exercise): experiences and preliminary results. J Med Educ.

[25] Chen YC (2010). Essential professional core competencies for nurses. Hu Li Za Zhi.

[26] National Organization of Nurse Practitioner Faculties (2013). Independent practice and the certified nurse practitioner - a white paper.

[27] National Organization of Nurse Practitioner Faculties (2015). Nurse Practitioner Core Competencies Content: A delineation of suggested content specific to the NP core competencies.

[28] Taiwan Nurses Association (2012). Clinical Ladder System program guideline for professional abilities of nursing staff.

[29] Canadian Nurses Association (2010). Canadian Nurse Practitioner-Core Competency Framework.

[30] Canadian Nurses Association (2010). Canadian Nurse Practitioner Core Competency Framework.

[31] Taiwan Nurses Association. Taiwan Nurses Association. 2018. https://www.twna.org.tw/frontend/un10_open/welcome.asp#. Accessed 10 Aug 2018.

[32] Norcini JJ, Blank LL, Arnold GK, Kimball HR (1995). The mini-CEX (clinical evaluation exercise): a preliminary investigation. Ann Intern Med.

[33] O'Connell J, Gardner G, Coyer F (2014). Beyond competencies: using a capability framework in developing practice standards for advanced practice nursing. J Adv Nurs.

[34] Linstone HA, Turoff M (1975). The Delphi method: techniques and applications.

[35] Cantillon P, Sargeant J (2008). Teaching Rounds: Giving feedback in clinical settings. BMJ.

[36] Bluestone J, Johnson P, Fullerton J, Carr C, Alderman J, Bon Temop J (2013). Effective in-service training design and delivery: evidence from an integrative literature review. Hum Resour Health.

[37] Liou SR, Tsai HM, Cheng CY (2014). Pregraduation clinical training program improves clinical competence of nurse students in Taiwan: an interventional study. J Nurs Educ Pract.

[38] Norcini JJ, Burch V (2007). Workplace-based assessment as an educational tool: AMEE guide no. 31. Med Teach..

[39] Lörwald Andrea C., Lahner Felicitas-Maria, Mooser Bettina, Perrig Martin, Widmer Matthias K., Greif Robert, Huwendiek Sören (2018). Influences on the implementation of Mini-CEX and DOPS for postgraduate medical trainees’ learning: A grounded theory study. Medical Teacher.

[40] Lee V, Brain K, Martin J (2019). From opening the ‘black box’ to looking behind the curtain: cognition and context in assessor-based judgements. Adv Health Sci Educ Theory Pract..

[41] Nursing Human Resources Survey (2015). Taiwan Union of Nurses Association.

[42] Lyn SL (2007). The role of the physical examination in clinical assessment: a useful skill for professional nursing. Pflege..

[43] Yousuf N. Mini clinical evaluation exercise: validity and feasibility evidences in literature. Educ Med J. 2012;4(1):e100-e107.

[44] Liao KC, Pu SJ, Liu MS, Yang CW, Kuo HP (2013). Development and implementation of a mini-clinical evaluation exercise (mini-CEX) program to assess the clinical competencies of internal medicine residents: from faculty development to curriculum evaluation. BMC Med Educ.

[45] Kak N, Burkhalter B, Cooper M-A. Measuring the competence of healthcare providers. QA Oper Res Issue Pap. 2001;2(1):1-28.

[46] Hawkins RE, Margolis MJ, Durning SJ, Norcini JJ (2010). Constructing a validity argument for the mini-clinical evaluation exercise: a review of the research. Acad Med.

[47] Van Der Vleuten CP (1996). The assessment of professional competence: developments, research and practical implications. Adv Health Sci Educ.

