# Supplementary material for: Development of a nursing-specific Mini-CEX and evaluation of the core competencies of new nurses in postgraduate year training programs in Taiwan
Source: BMC Med Educ. 2019 Jul 18;19:270. doi: 10.1186/s12909-019-1705-9 (PMC6639917; doi:10.1186/s12909-019-1705-9)
Supplement: Supplementary file 1 — Mini-Clinical Evaluation Exercise (Mini-CEX) for Nurses. (DOCX 25 kb) [file 12909_2019_1705_MOESM1_ESM.docx]

Additional file 1:

Mini-Clinical Evaluation Exercise (Mini-CEX) for Nurses

| Evaluator： | | | | | | Date： / / / | | | | | |  |
| --- | --- | --- | --- | --- | --- | --- | --- | --- | --- | --- | --- | --- |
| Trainee： | | | | | | Training duration：  (months) | | | | | |  |
| Patient’s diagnosis： | | | | | | | | | | | |  |
| Location：□Ward □Treatment room □Others  Patient information: Age： Gender：□Male □Female  □New patient □Returning patient | | | | | | | | | | | |  |
| Grading Criteria | Not Applicable | Grading | | | | | | | | | | |
|  |  | Unsatisfactory | | | Satisfactory | | | | Excellent | | | |
|  |  | 1 | 2 | 3 | 4 | | 5 | 6 | 7 | 8 | 9 | |
| History Taking |  |  |  |  |  | |  |  |  |  |  | |
| Physical Examination |  |  |  |  |  | |  |  |  |  |  | |
| Intervention/  Therapeutic Skills |  |  |  |  |  | |  |  |  |  |  | |
| Counseling Skills |  |  |  |  |  | |  |  |  |  |  | |
| Nursing Professionalism |  |  |  |  |  | |  |  |  |  |  | |
| Organization/  Efficiency |  |  |  |  |  | |  |  |  |  |  | |
| Overall |  |  |  |  |  | |  |  |  |  |  | |

Duration: Observation time: /min Feedback time: /min

Other comments：

Evaluator： Trainee：

Evaluation Dimensions:

History Taking

□ Perform a focused health assessment and/or an advanced comprehensive health assessment, using and adapting assessment tools and techniques based on patient needs and relevance to the patient’s stage of life.

□ Perform a complete or focused health history appropriate to the patient’s situation, including physical, psychosocial, emotional, ethnic, cultural, and spiritual dimensions of health.

□ Communicate with patients about health assessment ﬁndings and/or diagnosis, including outcomes and prognosis.

□ Maintain eye contact and proper body language while exercising good listening and questioning skills in order to convey empathy.

Physical Examination

□ Perform a complete or focused physical examination, in order to identify and interpret normal and abnormal ﬁndings as appropriate to patient presentation.

□ Order and/or perform screening and diagnostic investigations, interpret results using evidence-informed clinical reasoning and critical inquiry, and assume responsibility for follow-up.

□ Anticipate and diagnose emergent, urgent, and life-threatening situations.

Intervention/Therapeutic Management

□ Determine care options and initiate therapeutic interventions in collaboration with patients, while considering patient perspectives, feasibility, and best outcomes.

□ Initiate interventions for the purpose of stabilizing patients in emergent, urgent, and life-threatening situations.

□ Perform invasive/non-invasive procedures for the clinical management and/or prevention of diseases, injuries, disorders or conditions.

□ Support, educate, coach, and counsel patients regarding diagnoses, prognoses, and self-management, including their personal responses to diseases, disorders, conditions, injuries, risk factors, lifestyle changes, and therapeutic interventions.

□ Formulate a differential diagnosis by using history and physical examination data.

□ Involve patients in decision making.

□ Prescribe pharmacotherapy based on the patient’s health history, disease, disorder, condition, stage of life, and individual circumstances.

□ Obtain patient’s informed consent before performing interventions/treatments, prepare appropriately for the intervention/treatment, perform the procedure skillfully, and properly complete post-procedure activities.

Counseling Skills

□ Counsel patients on medication therapy regarding beneﬁts, potential side effects, interactions, importance of compliance, and recommended follow-up.

□ Assess the level of comprehension of families and patients when explaining issues in consultation or discussing treatment methods in order to determine what constitutes appropriate participation in the decision-making process.

□ Provide health promotion consultation services and conduct individual and group health education to assist patients discharged from the hospital.

Nursing Professionalism

□ Practice in accordance with federal and provincial/territorial legislation, professional and ethical standards, and policy relevant to nursing practice.

□ Adhere to federal and provincial/territorial legislation, policies and standards related to privacy, documentation, and information management (this applies to verbal, written, or electronic records).

□ Engage in ongoing professional development and accept personal responsibility for maintaining professional competency.

□ Collaborate with members of the health-care team to provide and promote inter-professional patient-centered care at the individual, organizational, and systems levels.

□ Consult with and/or refer patients to other health-care providers at any point in the care continuum when the patient’s condition is not within the nurse’s scope of practice or the individual nurse’s competency.

□ Display respect and concern while and interacting whole-heartedly with the patient.

□ Show proper sensitivity and professionalism towards the patient’s pain, disability, and background.

Organization/Efficiency

□ Integrate the principles of resource allocation and cost-effectiveness into clinical decision making.

□ Evaluate and solve problems with proper reasoning and logic.

Overall Clinical Competence

□ Introduce self and call patients by name when beginning/concluding the patient interaction.

□ Display professional mannerisms and appearance; maintain good eye contact, body language, and facial expressions; and speak in a suitable tone of voice with appropriate use of silence.
